# The Global Transmission and Control of Influenza

**DOI:** 10.1371/journal.pone.0019515

**Published:** 2011-05-06

**Authors:** Eben Kenah, Dennis L. Chao, Laura Matrajt, M. Elizabeth Halloran, Ira M. Longini

**Affiliations:** 1 Center for Statistics and Quantitative Infectious Diseases, Vaccine and Infectious Disease Division, Fred Hutchinson Cancer Research Center, Seattle, Washington, United States of America; 2 Department of Biostatistics, School of Public Health, University of Washington, Seattle, Washington, United States of America; 3 Department of Applied Mathematics, University of Washington, Seattle, Washington, United States of America; University of Hong Kong, Hong Kong

## Abstract

New strains of influenza spread around the globe via the movement of infected individuals. The global dynamics of influenza are complicated by different patterns of influenza seasonality in different regions of the world. We have released an open-source stochastic mathematical model of the spread of influenza across 321 major, strategically located cities of the world. Influenza is transmitted between cities via infected airline passengers. Seasonality is simulated by increasing the transmissibility in each city at the times of the year when influenza has been observed to be most prevalent. The spatiotemporal spread of pandemic influenza can be understood through clusters of global transmission and links between them, which we identify using the epidemic percolation network (EPN) of the model. We use the model to explain the observed global pattern of spread for pandemic influenza A(H1N1) 2009–2010 (pandemic H1N1 2009) and to examine possible global patterns of spread for future pandemics depending on the origin of pandemic spread, time of year of emergence, and basic reproductive number (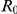). We also use the model to investigate the effectiveness of a plausible global distribution of vaccine for various pandemic scenarios. For pandemic H1N1 2009, we show that the biggest impact of vaccination was in the temperate northern hemisphere. For pandemics starting in the temperate northern hemisphere in May or April, vaccination would have little effect in the temperate southern hemisphere and a small effect in the tropics. With the increasing interconnectedness of the world's population, we must take a global view of infectious disease transmission. Our open-source, computationally simple model can help public health officials plan for the next pandemic as well as deal with interpandemic influenza.

## Introduction

Air travel has greatly accelerated the spread of influenza and other diseases transmitted by person-to-person contact. Countries with a higher volume of airline travel to and from Mexico experienced earlier outbreaks of pandemic H1N1 2009 [1], [2]. Mathematical and computer models including a global transportation network have been used to explore the spread of pandemic influenza [3]–[5]. However, the transportation network alone is not sufficient to predict the dynamics of an influenza pandemic.

Influenza has long been observed to peak in the winter months in the temperate northern hemisphere (i.e., north of the Tropic of Cancer) and temperate southern hemisphere (i.e., south of the Tropic of Capricorn) [6]. However, seasonality of influenza has not been sufficiently studied in the tropics, where it has been observed to peak during the rainy season or have no distinct seasonality [7], [8]. Previous models used simple functions to increase transmission during the winters of the temperate northern and temperate southern hemispheres but assumed constant transmissibility in the tropics (the region between the Tropic of Cancer and the Tropic of Capricorn). Though these models can replicate the annual peaks of influenza in the winters of the highly populated temperate northern and the less populated temperate southern hemispheres, they may not accurately reflect the dynamics in the tropics, an important region which may be the source of new pandemic influenza strains [9]–[11]. Our model attempts to model the seasonality of influenza in the tropics with greater accuracy and to understand the implications of influenza dynamics in the tropics for the public health response to a future pandemic.

Our model includes 321 major cities on six continents, the airline travel among them, and influenza seasonality data when available. This model includes more detailed within-host influenza dynamics, more detailed influenza vaccine behavior, and more detailed seasonality data for tropical regions than other models. Nonetheless, it is a relatively simple model that does not require specialized computing resources to use. Since it is open source, it can be used by anyone in the research or public health communities. Here, we use the model to explore the timing and spread of influenza on the global scale as a function of transmissibility (*i.e.*, the basic reproductive number, 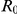), the origin of pandemic spread, and the time of year of emergence. Then, assuming that it takes roughly six months to make and to distribute vaccine, we investigate the effectiveness of the currently recommended vaccination strategies.

## Methods

### Mathematical Model

The model has two layers: a set of within-city models and a global model that links them through the air transportation network. We first describe the within-city model and then the global model. It is written in Python 2.6.2 ( http://www.python.org) using the SciPy and NumPy packages [12]. The code is available at http://www.csquid.org/software/globalmodel/.

Within each city, susceptibles are divided into subpopulations and risk groups. Subpopulation membership is used to determine transmission probabilities, and risk group membership is used to determine morbidity and mortality. In our simulations, we have two subpopulations, children (age 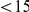 years) and adults (age 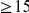 years), each with two risk groups (low and high risk). Since we focus on influenza transmission rather than morbidity and mortality, there is only a single risk group. The population of each city is divided into susceptible, infectious, and removed compartments, which have subcompartments to keep track of subpopulation, risk group, vaccination status, and symptom status (Text S1). Upon infection, each person is assigned to become asymptomatic or symptomatic. He or she is then assigned uniformly at random to one of the six viral load trajectories (Text S1 and Figure S1). On all trajectories, infection lasts six days but infectiousness varies with symptom status and viral load. In our simulations, infected persons become symptomatic with probability 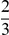, and asymptomatic infecteds are half as infectious as symptomatic ones. Infected persons transmit infection according to a next-generation matrix scaled to achieve a within-city 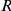, the reproductive number that is 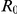 during influenza season and may be lower at other times of the year according to local influenza seasonality (Text S1). In our simulations, the transmission probabilities are tuned to obtain a next-generation matrix proportional that from [13], where the child-to-child transmission is 1.8, adult-to-adult transmission is 0.2, and the child-to-adult and adult-to-child transmissions are 0.5 (see Eqn 9 in Text S1). This ensures that child-child influenza transmission is most intense, adult-adult transmission is least intense, and child-adult and adult-child transmission are intermediate. Using all of this information and the effects of vaccination (described below), a system of discrete time and state-space stochastic equations (Text S1) governs spread of influenza within cities in one-day time steps. Major parameters are summarized in Table S1.

In the model, vaccination can reduce susceptibility to infection (by 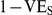 per infectious contact), infectiousness following infection (by 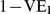), and the probability of becoming symptomatic after infection (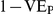). In our simulations, we use vaccine efficacy estimates for a well-matched seasonal influenza vaccine: 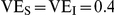 and 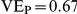, in accordance with [14]. These efficacies are not reached immediately upon vaccination, and we define the *vaccine efficacy ratio* function to be the proportion of full vaccine efficacy attained 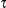 days after vaccination. As in [15], this function is governed by three parameters: 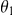 determines the shape of the increase in vaccine efficacy during the first 13 days, level is the maximum vaccine efficacy achieved after the first dose, and 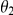 determines the shape of the increase in vaccine efficacy after the second dose (which is assumed to be given 21 days after the first dose). The underlying vaccine efficacy ratio function is:
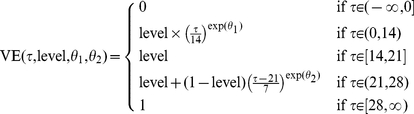
(1)


**Figure 1:**
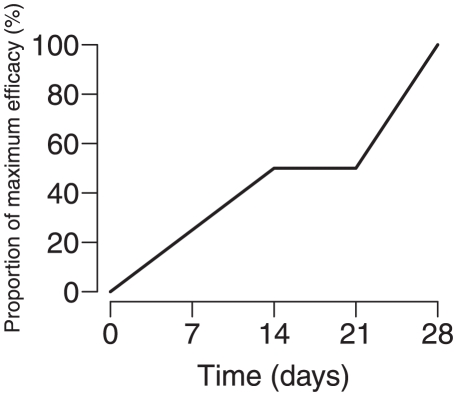
Vaccine efficacy over time. Vaccine efficacy rises over time, reaching maximum efficacy after 28 days. The model assumes that all individuals who receive their first dose vaccine will receive a second exactly 21 days later.

In the intervals 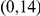 and 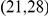, the function is concave for negative 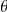, convex for positive 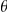, and linear for 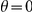 (Figure 1). 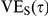, 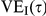, and 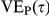 are obtained by multiplying 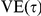 by the 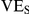, 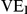, and 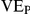, respectively. In our simulations, we use 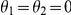, 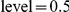.

The global model links together the 321 within-city models, allows travel between them, and controls seasonality. It records the total number of susceptibles, incident infections, infections, and recovereds in each time step, and (optionally) can store a matrix for each time step showing the number of travelers from each city to every other city. All cities are divided into two age groups: 0–14 years old and 15+ years old. The proportion of the population in each age group in each city is determined by the proportion of the population under 15 in the corresponding country [16]. All cities are assumed to have the same next-generation matrix at peak seasonal transmission. For each city 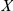, we have the average number of persons who travel to each other city in the model per day. We divide this by the population of 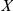 to get a probability of traveling from 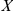 to each other city in the model at each time step (Text S1). Symptomatic individuals are 75% less likely to travel ( see sensitivity analysis in Figure S4). For efficiency, only infected travelers are tracked in the model. Infected visitors to a city are put into the infected compartment corresponding to their vaccination time, symptom status, viral load trajectory, and day of illness. They progress through the infected compartments and travel to other cities just like the other infected persons in the destination city. Upon recovery, they return immediately to their home city. Because of the short infectious period of influenza, we assumed that infected travelers would not have the opportunity to return before recovering. A city's population may experience small and temporary fluctuations in size because of travel, but the number of travelers is much smaller than the population size.

### Seasonality of influenza in the model

**Figure 2:**
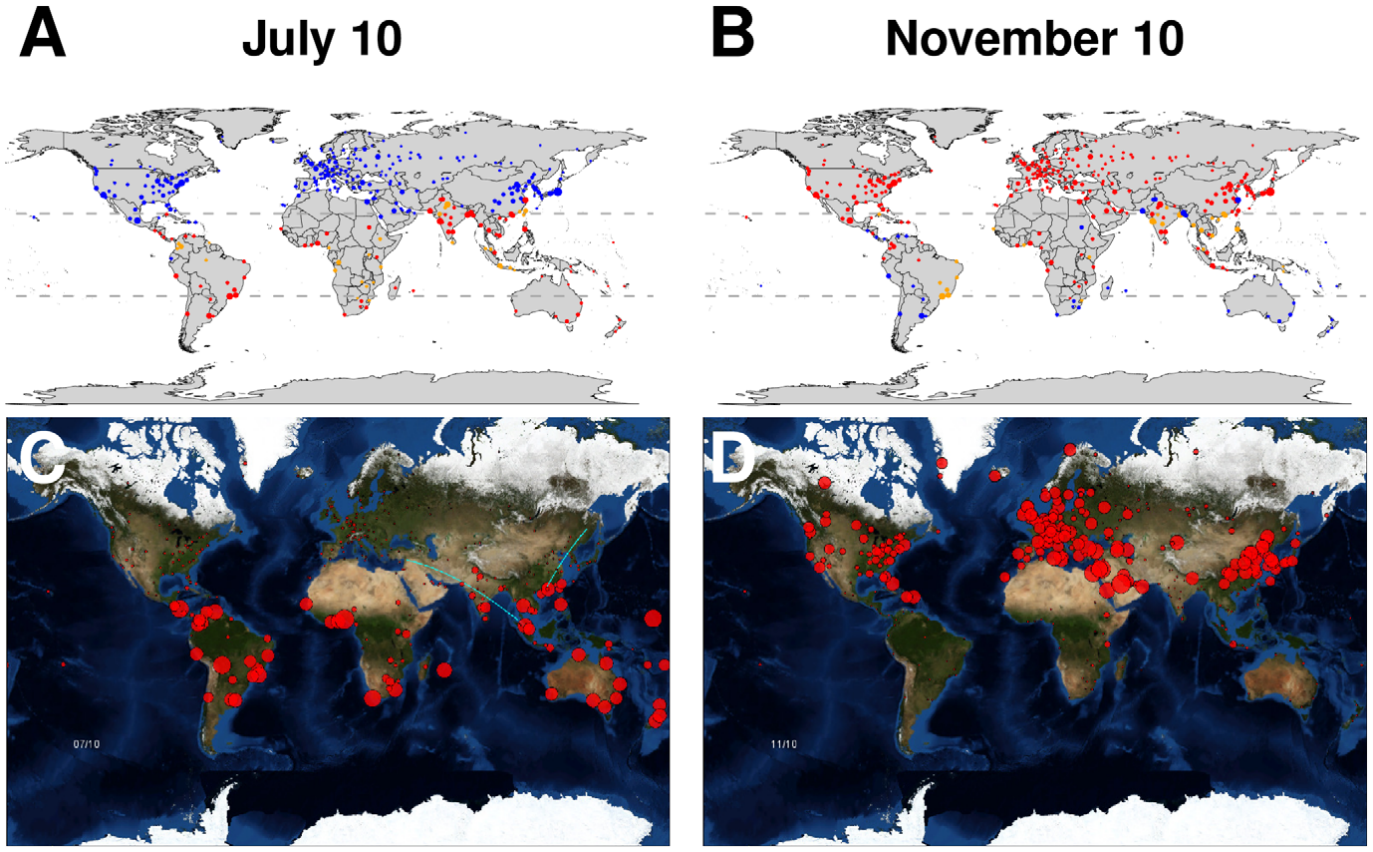
Seasonality in the model affects epidemic dynamics. The maps in the top row depict the relative transmissibility of influenza in the model on (A) July 10 and (B) November 10. Each city in the model is plotted with a dot size proportional to the city's population and colored red when influenza is highly transmissible, blue when influenza is least transmissible, and orange for intermediate levels of transmissibility. The influenza season in the temperate northern and temperate southern hemispheres occurs during their respective winters, hence the large proportion of red dots in the south on July 10 and red dots in the north on November 10. The Tropics of Cancer and Capricorn are plotted as dashed horizontal lines. Seasonality in the tropics does not follow this pattern, and may have multiple peaks, often corresponding to the rainy season. The maps in the bottom row show the prevalence of influenza in each city in a simulation in which the pandemic began in Mexico City on April 1. The size of each red dot is proportional to the prevalence of influenza in each city. (C) In July, prevalence is high in several cities in South America. (D) In November, prevalence is high across the temperate northern temperate regions. Large epidemics can only occur when the seasonal transmissibility in a city permits.

Transmissibility for each city rises and falls in an annual cycle in the model (Figure 2). In the model, a country is always either in-season and transmission is high (i.e., 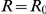) or out-of-season (i.e., 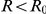). For cities north of the Tropic of Cancer, influenza transmission was assumed to be high (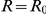) from September 15 to June 1 each year and low otherwise (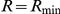), except for cities that are known to deviate from this pattern. Likewise, influenza transmission was assumed to be high from April 15 to October 15 for cities south of the Tropic of Capricorn (Figure S2). For temperate regions, we assumed that transmission was 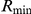 outside of influenza season. For regions known to have year-round transmission, we assumed that transmission was 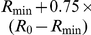 outside of influenza season. In other parts of the tropics, we assumed that transmission was 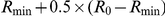 outside of influenza season.

Seasonality of influenza transmission is likely to be caused by a variety of factors, from annual weather cycles to social factors [17]. We collected data on influenza season in various parts of the world from the literature (Table S2). For large regions, influenza epidemics peak about two months after they start [18], so if the timing of the peak of influenza is known for a country, then we assumed that the influenza season started about two months earlier. If epidemic curves were shown and there was an obvious peak of influenza activity, we defined the influenza season to cover the peak as well as the periods elevated activity before and after the peak. For regions in the tropics for which there was no influenza activity data available, we assumed that influenza season coincided with the rainy season. A few countries, such as China, India, and Brazil, are known to have different influenza seasons in different regions, and we tried to infer the season for each city when possible. The seasonality used for each city in the model is summarized in Figure S3.

### Mapping global influenza transmission

To better understand the global transmission of influenza, we identified clusters of cities within which transmission occurs rapidly. To do this, we started with the epidemic percolation network (EPN) of the global model (Text S2). The EPN is a directed random graph that represents the final outcomes of a stochastic epidemic model [19], [20]. Informally, the EPN is a graph where we draw directed edges from each person 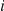 to all persons 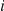 would infect if the population were entirely susceptible. If an epidemic begins with the infection of person 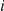, all persons who can be reached from 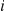 by following a series of edges—the out-component of node 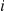 in the EPN—will be infected. Thus, the EPN gives us a final outcome of the epidemic model for any given set of initial infections.

The EPN for the entire global model would include hundreds of millions of nodes and edges. To map the global spread of infection, we collapsed the full EPN into a city-to-city EPN with a directed edge from each city 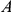 to all cities that can be reached directly from 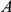 (Text S2). The weight of each edge 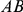 is proportional to the expected number of directed edges in the EPN pointing from persons in city 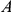 to persons in city 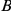, assuming that all cities are transmitting at their peak seasonal 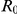. This resulted in a network with 321 nodes and 53,534 weighted links. We simplified this network with an information-theoretic clustering algorithm [21] based on finding a two-level code that minimizes the expected code length required to describe the path of a random walker on the city-to-city EPN (Text S2). We used the Map Generator software package at www.mapequation.org [22] to perform the clustering algorithm and generate the map.

## Results

The model fit the observed spread of pandemic H1N1 2009 when the simulated epidemic was started with 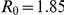 in Mexico City in late March 2009 (see Movie S1, Table S4, and Text S3). In a sensitivity analysis, we found that later epidemic start dates required higher values of 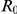 for the pandemic to spread to the northern hemisphere at the appropriate time (Figure S5). Figure 2C shows a snapshot of the simulated global spread on July 10, 2009, as the pandemic had swung to the temperate southern hemisphere as well as the tropics, and Figure 2D shows the state of the pandemic on November 10, 2009, as the pandemic was just passing peak activity in much of the temperate northern hemisphere. In addition, we modeled global transmission of a strain for influenza more like the Hong Kong influenza A(H3N2) pandemic of 1968–1969 with 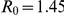 (see Movie S2, Table S5, and Text S3). The model did not fit the observed data as well, probably because the pandemic took multiple seasons to reach certain regions (Figure S6).

### Network structure of global influenza transmission

**Figure 3:**
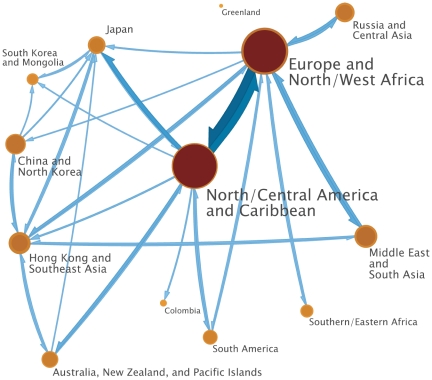
Map showing influenza transmission clusters. Clusters represent groups of cities within which transmission of influenza is rapid; transmission between clusters is slower. The map shows all 13 clusters and the 36 directed edges across which the most inter-cluster transmission occurs, which account for 90% of all inter-cluster influenza transmission. The area of each cluster is proportional to the steady-state proportion of time a random walker on the city-to-city EPN spends in the cluster. The proportion of each cluster contained in its border ring equals the probability that a random walker within the cluster jumps to a city in a different cluster, so the proportion contained in the interior is equal to the probability that a random walker within the cluster jumps to another city in the cluster. The width of each edge is proportional to the steady-state proportion of jumps between clusters that cross it. For emphasis, the color of cluster interiors and border rings gets darker with increasing area and the color of the edges gets darker with increasing width.

The clustering algorithm identified 13 clusters connected by 146 directed edges. Table 1 summarizes characteristics of the clusters, and Table S3 lists the cities in each cluster. “Flow” is the steady-state proportion of time that a random walker on the city-to-city EPN spends within the cluster, “outflow” is the steady-state probability that a random walker within the cluster jumps to a city in a different cluster. To measure the relative importance of the cities within each cluster to the global transmission of influenza, we divided the cluster's flow by the number of cities it contains, normalizing so the average value over all clusters equals one. This is called the “per-city flow” in the table. Figure 3 shows the 13 nodes in their approximate geographical locations and the 36 edges across which the most inter-cluster influenza transmission occurs, which account for 90% of all transmission between clusters.

**Table 1:** Global transmission cluster characteristics.

Cluster	# of cities	Flow^1^	Outflow^2^	Flow per city^3^
Europe and North/West Africa	85	0.309	0.187	1.30
North/Central America and Caribbean	64	0.296	0.106	1.65
Middle East and South Asia	34	0.071	0.324	0.74
Hong Kong and Southeast Asia	16	0.065	0.459	1.45
China and North Korea	24	0.056	0.231	0.84
Russia and Central Asia	41	0.051	0.403	0.44
Japan	7	0.041	0.406	2.10
Australia, New Zealand, and Pacific Islands	15	0.037	0.254	0.88
South America	12	0.028	0.257	0.84
Southern/Eastern Africa	12	0.022	0.360	0.64
South Korea and Mongolia	5	0.017	0.473	1.24
Colombia	5	0.005	0.470	0.63
Greenland	2	0.001	0.683	0.24

1 Steady-state proportion of steps spent in the cluster by a random walker.

2 Steady-state probability that a random walker in the cluster jumps to a different cluster.

3 Flow divided by number of cities, normalized so the average value equals one.

The map captures several important features of global spread of influenza. The major population centers in the temperate northern hemisphere are highly connected, resulting in a narrow epidemic curve with a single peak. Connections between clusters in the tropics and the temperate southern hemisphere are not as dense, resulting in wider epidemic curves with multiple peaks. The Hong Kong and Southeast Asia cluster plays a larger role in the spread of influenza than would be expected based on the number of cities it contains. The North/Central America+Caribbean and Europe+North/West Africa clusters have high flow per city but relatively low outflow probabilities. The Hong Kong and Southeast Asia cluster has both high flow per city and a high outflow probability. Its tropical location allows it to serve as a bridge between the Northern and Southern Hemispheres. The map indicates that pandemics starting within season in the temperate northern hemisphere, North and West Africa, or the Caribbean would quickly spread throughout those regions but diffuse much more slowly to other parts of the globe. Epidemics originating in China are linked to North America and Europe primarily through Japan and Southeast Asia.

### Global patterns of pandemic influenza spread

**Figure 4:**
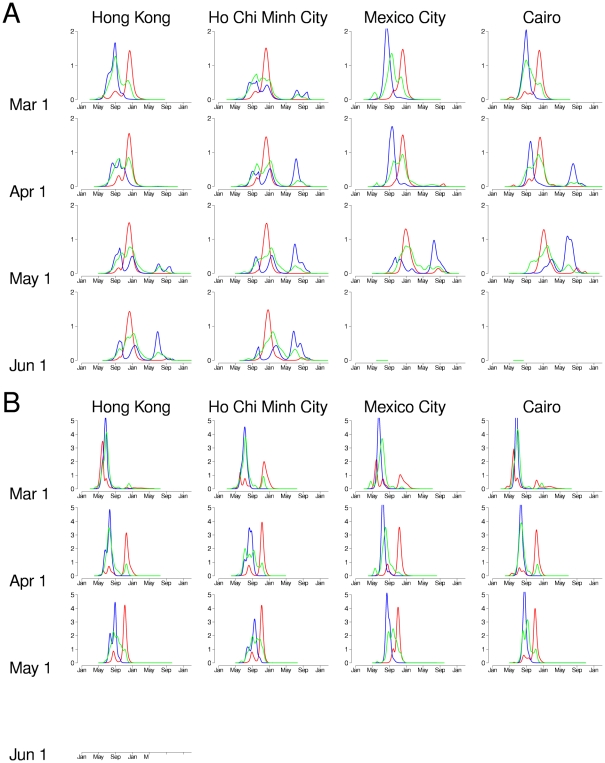
Prevalence of influenza over time in simulated pandemics with various starting locations, dates, and transmissibilities. In each plot, the infection prevalence (%) for the cities in the three regions of the globe are plotted: North (cities north of the Tropic of Cancer, in red), South (cities south of the Tropic of Capricorn, in blue), and tropics (cities between the two tropics, in green). Each plot shows the results from a single simulation. (A) Simulated pandemics with 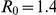 were started in Hong Kong (first column), Ho Chi Minh City (second column), Mexico City (third column), and Cairo (fourth column). The epidemics were started on March 1, April 1, May 1, and June 1 and plotted in the first, second, third, and fourth rows, respectively. (B) Plots of simulated pandemics with 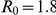, organized as in panel A. Note that the y-axis has a different scale than panel A.

To investigate the most plausible global patterns for pandemic influenza spread, we model the initial outbreak to occur at different key geographic locations, times of the year, and values of 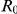. In Figure 4, we show plots of the global spread for pandemics starting in Hong Kong, Ho Chi Minh City, Cairo and Mexico City. Hong Kong was the first city to experience a large epidemic in the Hong Kong influenza pandemic in 1968. Both Egypt and Vietnam have experienced considerable avian influenza A(H5,N1) human cases where reassortment events could lead to a pandemic strain.

The scenario most similar to the Hong Kong pandemic of 1968–1969 is shown in the plot for Hong Kong starting on June 1 in Figure 4A. In this case, we see two peaks in the temperate southern hemisphere countries because the first wave out of Hong Kong does not go to completion before the end of the influenza season in many of these countries (Movie S2). If initial spread comes out of Hong Kong earlier in the year (i.e., March or April), then most of the temperate southern hemisphere peaks during the winter of the first year. In all these cases, a single peak occurs in December in the temperate northern hemisphere countries. The epidemics in the tropics tend to peak in multiple clusters between August and January. A similar pattern occurs if the pandemic starts in Ho Chi Minh City rather than Hong Kong.

For pandemics starting in Mexico City or Cairo, with 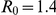, two peaks occur in the temperate southern hemisphere if the pandemic starts on April 1, but only one peak occurs if the pandemic starts on March 1. The epidemics in the temperate northern hemisphere and the tropics are roughly the same as when the pandemic started in Hong Kong or Ho Chi Minh City. If the pandemic strain starts spreading on May 1 or June 1, there is no pandemic at all. In this case, we are past the temperate northern hemisphere influenza season and infected travelers arrive too late in the temperate southern hemisphere influenza season to sustain transmission there.

Figure 4B shows simulations of pandemics having initial spread in the same four cities for a more transmissible virus with 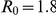. In these cases, the pandemic is much larger and faster than when 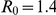 and the patterns of spread are similar regardless of when and where the pandemic spread starts. The case where pandemic spread starts in Mexico City on April 1 is closest to the pandemic H1N1 2009 situation. In this case, there is only a single first peak in the temperate southern hemisphere in July and a large peak in the temperate northern hemisphere in late October. As in the case when 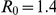, there is no subsequent pandemic when spread starts in Mexico City on June 1.

### Effect of a global vaccination strategy

For our modeling, we assume that pandemic vaccine is available 180 days after the appearance of the pandemic strain. For pandemic H1N1 2009, substantial quantities of vaccine became available in October, 2009, roughly five to six months after the recognition of the pandemic strain in late April, 2009. In the US, the epidemic peaked in October, just as the vaccine was arriving. We assumed that all vaccine was delivered and administered at once, and more realistic rollouts would result in a slower and possibly less efficacious global mass vaccination campaign.

We used the per capita GDP from 2007 [23] to determine how much vaccine each country would be able to obtain. Wealthy countries (per capita GDP 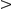
$25,000 in year 2000 dollars) cover 50% of their populations. Other developed countries (per capita GDP 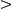
$10,000) cover 25% of their populations. The remaining countries cover 10%, many relying on the World Health Organization's vaccine distribution plan. See Table S6 for a summary of vaccine coverage in the model for individual countries.

To reduce mortality and morbidity, vaccine should first be distributed to children and individuals at high risk of complications from influenza infection [24], [25]. We assume that 10% of children are and 17% of adults are at high risk [26]. In the simulations, we prioritize high-risk children, followed by high-risk adults, healthy children, then healthy adults. We assume that a maximum of 50% of any group will get vaccinated. Therefore, if a country can cover 50% of its population, then 50% of each of these risk groups is covered. If a country can cover 25% of its population, then 50% of the high-risk children and adults are covered, 50% of the healthy children, and about 10–13% of healthy adults. If a country can cover 10% of its population, then 50% of the high-risk children and adults are covered, about 5% of healthy children, and no healthy adults. This strategy results in lower overall attack rates as compared to a strategy in which everyone has the same priority (Figure S8).

Table 2 shows the results for such a vaccination campaign for several of the pandemic scenarios shown in Figure 4. The row for the pandemic starting in Mexico City on April 1, with 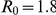 gives the scenario closest to pandemic H1N1 2009. In this case, the model predicts about 1.2 billion eventual influenza illnesses with no vaccination and about 930 million had vaccination been carried out as described above. The biggest impact of vaccination would have been in the temperate northern hemisphere, reducing the illness attack rate from 16% to 9%. Such a vaccination campaign would have little effect on the epidemic in the temperate southern hemisphere, and a small effect in the tropics. Movie S3 gives a dynamic view of the effect of such vaccination. This vaccination plan has the biggest beneficial effect in the temperate northern hemisphere if the pandemic strain begins spread from Mexico City or Cairo in March or April. For the temperate southern hemisphere, this vaccination strategy would have the biggest beneficial effect if the 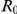 is lower, at 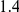, and if the beginning of pandemic spread is in Hong Kong in May or June, or Cairo in April. In general, the tropics benefit most only when the other regions gain a benefit as well.

**Table 2:** Potential global impact of mass influenza vaccination (averages from 10 simulations for each scenario).

					illness attack rate, %			
Origin	R 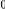	Start date	Intervention	Ill, Millions	Total	North	South	Tropics
Hong Kong	1.4	May	Baseline	745	11	10	11	13
			Vaccination	432	6	5	6	10
		Jun	Baseline	732	11	10	11	13
			Vaccination	541	8	7	6	10
	1.8	May	Baseline	1234	18	16	19	22
			Vaccination	1159	17	14	18	22
		Jun	Baseline	1214	18	16	17	22
			Vaccination	1205	18	16	16	21
Mexico City	1.4	Mar	Baseline	762	11	10	13	14
			Vaccination	400	6	3	12	10
		Apr	Baseline	738	11	10	10	13
			Vaccination	355	5	3	9	9
	1.8	Mar	Baseline	1168	17	14	20	22
			Vaccination	1057	16	12	20	22
		Apr	Baseline	1232	18	16	20	23
			Vaccination	926	14	9	20	22
Cairo	1.4	Mar	Baseline	782	11	10	12	14
			Vaccination	429	6	4	10	11
		Apr	Baseline	754	11	10	10	13
			Vaccination	384	6	3	7	10
	1.8	Mar	Baseline	1133	17	14	20	22
			Vaccination	1028	15	11	20	22
		Apr	Baseline	1232	18	16	20	23
			Vaccination	922	14	9	20	22

## Discussion

Our analysis of the global spread of pandemic influenza gives some insight into the spread of genetically drifting interpandemic strains of influenza. The cities in our model include about 620 million individuals, or about one tenth of the world's population. These geographically distributed major population centers should be enough to represent the overall dynamics of a global epidemic, in which influenza strains travel via infected passengers from epidemic regions to those just coming into influenza season. Previous modeling studies have shown that air travel governs the rapid dynamics of epidemic spread around the globe, and that other modes of transport govern the slower local regional diffusion of disease [27]–[29]. Our more detailed treatment of influenza seasonality in the tropics contributes greatly to the realism of the model without increasing its computational complexity.

Although SE Asia has often been the source of new strains of seasonal influenza, the next pandemic may arise in other parts of the world, as was demonstrated by pandemic H1N1 2009 in early 2009. One of the more alarming scenarios would be a newly reassorted H1N1/H5N1 influenza with high transmissibility and virulence. Therefore, we considered regions with potential person-to-person transmission of H5N1 [8].

The cluster map of global influenza transmission in Figure 3 helps explain several important features of our simulation results. In all of the scenarios we simulated, pandemics peaked in the temperate northern hemisphere during the fall/winter of the first year (Figure 4), as has been observed historically. The temperate northern hemisphere is highly connected by air travel and may share a common winter influenza season, so influenza prevalence peaks across much of this region appear to be synchronized [30]. In the temperate southern hemisphere, epidemics may peak in the fall/winter of either (or both) the first or second year, depending on when the epidemic starts and how transmissible it is. The tropics, which do not have a single unifying influenza season and are less densely connected, has unsynchronized epidemic peaks. Epidemics with a high 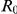 are likely to burn out in one season, while those with lower transmissibility may take multiple seasons to reach the more remote parts of the world. In particular, South America is not well-connected to most of the world in our model (Figure 3). Its strongest links are with the North and Central America cluster and the Europe and North/West Africa cluster, where most cities are out of season during the southern influenza season. This path of transmission from Asia to the temperate northern hemisphere, and much later to South America agrees with phylogenetic analyses of influenza strains around the world [11], [31]. How these strains evolve each season after they leave the tropics is open to debate [10], [31], [32].

To duplicate the observed global dynamics for pandemic H1N1 2009, we set the value of 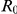 to 1.8, at the higher end of the estimated range of 1.3–1.7 from early spread in Mexico and the US [15] but consistent with another global model of pandemic H1N1 [4]. Our model predicts that parts of the globe already invaded by pandemic H1N1 2009 will not experience substantial further epidemics (see panel for Mexico City and April in Figure 4B), unless the virus begins genetic drift under immune pressure. Following pandemic years, increasing levels of population immunity change the age-specific transmission patterns of circulating strains. Further study will be needed to build reliable global simulation models of interpandemic strains. In addition, our models predicted that the temperate northern hemisphere would have had considerable reduction in the influenza illness attack rates had vaccine been distributed in the quantities indicated, i.e., rapid 50% coverage, on October 1. However, that was not the case. In the US, small quantities of vaccine arrived in early October, ramping up to about 20% coverage by December, 2009. We estimate that the effect of vaccination in the US reduced the illness attack rate from about 23% to about 20%. Thus, vaccine would have to be delivered in a more timely fashion and with higher coverage in the US and other countries to have the effectiveness predicted by our model.

The model uses many simplifying assumptions to be tractable, and it may be misspecified in ways that bias our results. Recent models have begun to incorporate more realistic networks of human movement, including ground transportation [4], [29]. The addition of commuting patterns do not substantially change the timing of the epidemic peaks [29], but this level of detail may be required to simulate the dynamics of epidemics at finer resolutions [28]. The fact that our model is open-source and computationally simple enough to run easily on a laptop makes it more accessible to the public health community than proprietary, computationally intensive models. Our model uses the same next-generation model in all cities. Regional differences in population structure and in the behavior of children and adults, including hygiene, socializing, and propensity to travel, may influence the global spread of influenza. We have performed a simple sensitivity analysis for age structure (see Figure S7), but this is an area that needs further exploration. We suspect that more accurate next-generation matrices in the cities of our model would increase the relative importance of influenza transmission in the tropics, which includes many countries with very young population. This, in turn, makes accurate modeling of seasonality even more crucial for obtaining realistic simulation results.

The factors that influence the seasonality of influenza are not well understood, so we used the observed influenza activity from past seasons to define periods of high transmissibility. One problem with this approach is that the model predictions do not take into account the conditions of a particular year. A more detailed model would allow seasons to be delayed or truncated by, for example, climate and school calendars [33], [34]. Although it would be conceptually easy add such conditions to the model, the amount and availability of required data are significant obstacles. Nonetheless, we believe that the tropical seasonality of influenza in our model is an important improvement on earlier efforts.

The single-strain model that we present here is suitable for pandemics, in which there is little pre-existing immunity in the population. However, the dynamics of seasonal influenza are determined by multiple competing strains, cross-protection, antigenic drift, and waning immunity. None of this is captured in our model, which may limit its use in planning a public health response to inter-pandemic influenza spread.

The transmission cluster map captured several important features of global influenza transmission, and we believe it is a new and useful way to understand the behavior of complex epidemic models. The clustering algorithm could be modified in several ways that might improve the identification of transmission clusters. The current algorithm was not designed specifically to understand infectious disease transmission, so it is insensitive to the effects of seasonality and to the population within each city. Improving the identification of transmission clusters and understanding their use in the design of global vaccination strategies are important extensions of the research presented here.

We investigated a likely global distribution of pandemic influenza vaccine within current possible constraints using an open-source model that we developed to capture the essential features of global influenza transmission while remaining computationally simple enough to be used by any researcher. We show that such strategies are marginally effective for certain regions of the planet depending on the location, timing and transmissibility of the new pandemic strain. The modeling structure and clustering algorithm used for Figure 3 could be used to develop optimal vaccine distribution if global strategies were possible for limited quantities of vaccine. This would be an important next step for the control of both pandemic influenza and interpandemic influenza, and it is a subject of future research and planning.

## Supporting Information

Movie S1
**Animation of a simulated pandemic H1N1 2009-like pandemic.** The simulation was initialized with 1,000 infected individuals in Mexico City on March 29 with 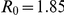. Red dots on the map indicate cities with infected individuals, with the size of the dot proportional to prevalence. Light blue arcs indicate that an infected person travels to a city with no infected individuals. On the right, infection prevalence is plotted for three regions: North (cities north of the Tropic of Cancer, in red), South (cities south of the Tropic of Capricorn, in blue), and tropics (cities between the two tropics, in green).(MPG)

Movie S2
**Animation of a simulated pandemic beginning in Hong Kong.** The simulation was initialized with 1,000 infected individuals in Hong Kong on June 1 with 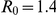.(MPG)

Movie S3
**Animation of simulated pandemics beginning in Mexico, with or without vaccination.** The simulation was initialized with 1,000 infected individuals in Mexico City on April 1 with 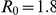. The top panels show a map and the prevalence of infection when there is no vaccine available, while the bottom panels correspond to the simulation in which vaccine was administered on September 1. In countries in which the per capita GDP was over $25,000 in 2007, 50% of the population was vaccinated. In countries in which the per capita GDP was less than $25,000 but over $10,000, 25% of the population was vaccinated. 10% of the population was vaccinated in the remaining countries.(MPG)

Figure S1 **The six viral load trajectories.** Data from [35], [36].
(EPS)

Figure S2 **The locations of the 321 cities in the global transportation network.** Dot size is proportional to population. Red points are north of the Tropic of Cancer, blue points are south of the Tropic of Capricorn, and green dots are between the two tropics.
(EPS)

Figure S3
**Influenza seasons in the model.** Each row of symbols represents the seasonality of a single city over the course of a year, with the exception of the first row, which represents all cities north of Lahore, Pakistan. Cities labeled in red are Northern (above Tropic of Cancer), those in green are in the tropics, and those in blue are Southern. Red triangles represent high transmissibility (influenza season) in a temperate region, 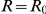. Blue triangles are low transmissibility (out-of-season) in a temperate region, 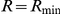. Orange triangles represent high transmissibility in a tropical region, 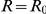. Green triangles are (relatively) low transmissibility in a tropical region, 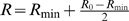. Magenta circles are moderate transmissibility in a tropical region, 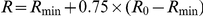.(EPS)

Figure S4
**Sensitivity of the model results to the symptomatic vs healthy travel ratio.** Top panel: The model was run for a pandemic H1N1-like scenario, starting on March 29 with 1,000 individuals infected with a strain with 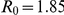. The symptomatic to healthy travel ratio was varied from 0% to 100%. For each value of this ratio, the simulation was run 10 times, and the epidemic peak for each country is plotted as a “+”. When the ratio is 0%, infected travelers only travel when they will not become symptomatic, which caused epidemics to peak later in countries. When the ratio is 100%, symptomatic travelers travel just as often as healthy individuals, causing epidemics to peak early in the southern hemisphere and late in the northern hemisphere. Bottom panel: The model was run using the 1968–69 air travel network and “Hong Kong”-like parameters (pandemic starting with 1,000 individuals in Hong Kong with 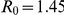 on May 24). The peak time for Sydney, Australia, was most affected by the symptomatic to healthy traveler ratio. At high values, the epidemic peaked during the first season, while at increasingly lower values the epidemic peak would occur more frequently during the second season.(EPS)

Figure S5
**Fitting the model to pandemic H1N1 2009.** Top panel: Estimates of R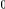 and the pandemic start date. We varied R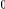 in increments of 0.05 and the pandemic start date (day on which 1,000 people are infected in Mexico City) in increments of one week. We ran the simulation once for each combination of values. The numbers in the plot are the 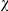-square values. Dots are yellow where 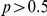, orange where 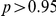, and red where 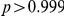. Bottom panel: Influenza prevalence in the model for the 2009–2010 H1N1 pandemic. We assumed that the pandemic started with 1,000 infected individuals in Mexico City on March 29 with R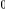 = 1.85. Model predictions are by city, so a country is the sum of its cities. The peak day for each country in a single simulation is in the legend.(EPS)

Figure S6
**Fitting the model to the 1968–69 Hong Kong pandemic.** Top panel: Estimates of R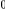 and the pandemic start date. We varied R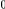 in increments of 0.05 and the pandemic start date (day on which 1,000 people are infected in Hong Kong) in increments of one week. We ran the simulation *twice* for each combination of values and chose the results with the smaller errors. The numbers in the plot are the Chi-square values. Dots are yellow where 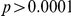 and orange where 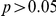. Bottom panel: Influenza prevalence in the model for the 1968–1969 pandemic. We assumed that the pandemic started with 1,000 infected individuals in Hong Kong on May 24 with R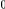 = 1.45.(EPS)

Figure S7
**Sensitivity of the model results to population age structure.** Top panel: The model was run for a pandemic H1N1-like scenario, starting on March 29 with 1,000 individuals infected with a strain with 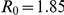. In the default scenario (in black), the fraction of children in the population of each country was based on [16]. In the alternative scenario (in red), the population was not divided into children and adults. The simulation was run 10 times for each scenario, and the epidemic peak for each country is plotted. Bottom panel: The model was run using the 1968–69 air travel network and “Hong Kong”-like parameters (pandemic starting with 1,000 individuals in Hong Kong with 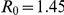 on May 24). The peak time for Sydney, Australia, was most affected by the symptomatic to healthy traveler ratio. At high values, the epidemic peaked during the first season, while at increasingly lower values the epidemic peak would occur more frequently during the second season.(EPS)

Figure S8
**The effect of vaccination on influenza prevalence in the model by hemisphere.** Top: For a 1968–1969-like pandemic, we assumed that it began with 1,000 infected individuals in Hong Kong on June 1 with R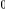 = 1.4 and vaccination occurred 180 days later in late November. Bottom: For a 2009-like pandemic, we assumed that the pandemic started with 1,000 infected individuals in Mexico City on April 1 with R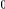 = 1.8 and vaccination occurred 180 days later in late September. Solid lines are for no vaccination, dashed lines plot “Universal” vaccination (everyone has the same priority), and dotted lines plot vaccination that prioritizes high risk individuals and children. The color of the line indicates region. Small differences between scenarios might be due to stochastic effects. Each plot shows a single stochastic realization.(EPS)

Table S1 **Model parameters.**
(PDF)

Table S2 **Influenza season data from the literature.**
(PDF)

Table S3 **Cities in the 13 transmission clusters from the global model, in decreasing order of flow.**
(PDF)

Table S4 **Observed and simulated pandemic H1N1 2009 epidemic peaks.** Observed data was from influenza A virology surveillance data from Flunet.
(PDF)

Table S5 **Observed and simulated 1968–1969 pandemic peaks.** Observed data is from [3].
(PDF)

Table S6 **Vaccine availability in different countries in the model.**
(PDF)

Text S1 Model.
(PDF)

Text S2 Mapping global influenza transmission.
(PDF)

Text S3 Pandemic simulations.
(PDF)
