# Supplementary material for: The Global Transmission and Control of Influenza
Source: PLoS One. 2011 May 6;6(5):e19515. doi: 10.1371/journal.pone.0019515 (PMC3089626; doi:10.1371/journal.pone.0019515)
Supplement: Table S1 — Model parameters. (PDF) [file pone.0019515.s012.pdf]

Table S1. Model parameters

Parameter	Value	Description	Source
$\mathsf{VE_S}$	0.4	Reduction in susceptibility from vaccination	[1]
$\mathsf{VE_I}$	0.4	Reduction in infectiousness from vaccination	[1]
$\mathsf{VE_P}$	0.667	Reduction in becoming symptomatic upon infection from vaccination	[1]
τ	28 days	Time for a vaccine to reach full efficacy	
R_0	1.4–1.85	Basic reproductive number in-season	
R_{min}	0.8	Reproductive number out-of-season	
m	0.5	ratio of asymptomatic to symptomatic infectiousness	
p_{symp}	0.67	probability of becoming symptomatic given infection	[2]
`sympTratio`	0.25	Ratio of travel probability of travel of symptomatic to asymptomatic	
v_i		Relative infectiousness on day i	[3–4]

[1] Basta NE, Halloran ME, Matrajt L, Longini IM Jr (2008) Estimating influenza vaccine efficacy from challenge and community-based study data. Am J Epidemiol 168: 1343–52.
[2] Carrat F, Vergu E, Ferguson NM, Lemaitre M, Cauchemez S, et al. (2008) Time lines of infection and disease in human influenza: a review of volunteer challenge studies. Am J Epidemiol 167: 775–85. [3] Murphy BR, Rennels MB, Douglas RG Jr, Betts RF, Couch RB, et al. (1980) Evaluation of influenza A/Hong Kong/123/77 (H1N1) ts-1A2 and cold-adapted recombinant viruses in seronegative adult volunteers. Infect Immun 29:348–55.
[4] Baccam P, Beauchemin C, Macken CA, Hayden FG, Perelson AS (2006) Kinetics of influenza A virus infection in humans. J Virol 80: 7590–9.
