# Supplementary material for: The Global Transmission and Control of Influenza
Source: PLoS One. 2011 May 6;6(5):e19515. doi: 10.1371/journal.pone.0019515 (PMC3089626; doi:10.1371/journal.pone.0019515)
Supplement: Table S2 — Influenza season data from the literature. (PDF) [file pone.0019515.s013.pdf]

Table S2. Influenza season data from the literature.

Country or city	Season	References
	temperate northern hemisphere	
Canada	mid-September to early June	[1]
Japan	November–April	[2, 3]
South Korea	January–April	[2]
South Korea	November–February	[3]
Iran	Oct–April, peak in Feb	[4]
Saudi Arabia	begins in Sept, peaks in Nov–Dec	[5]
	temperate southern hemisphere	
Australia	April–Oct	[2, 3]
	tropics and other regions	
China	winter peak in N, summer (but year-round) in S	[6]
Northern China	November–February peak	[3]
Shanghai, China	July–September, may have smaller peak in March	[2]
Guangdong, China	March–October	[2]
Hong Kong, China	April–August, may have smaller peak in March	[2]
Hong Kong	January–March, July–August (small peak), year-round	[7]
Hong Kong	April, year-round	[8]
Taiwan	December–March, year-round	[9]
Mongolia	November–March	[3]
Pune, India	June–September	[10, 3]
Delhi, India	September–March, activity throughout the year	[3]
Kolkata, India	May–September	[11]
Bangladesh	May–September	[12]
Pakistan	July–October peak	[2]
Thailand	May–October, activity all year	[2]
Malaysia	April–June, activity all year	[2]
Singapore	March–July, smaller peak in Oct–Jan, activity all year	[2, 3]
Hanoi, Vietnam	peak in June–August, year-round	[13]
Cambodia	June–December	[14, 15]
Philippines	June–September, smaller peak in Dec–Jan	[3]
Indonesia	December–January peak	[16]
Papua New Guinea	Jul–Sept peak, year-round	[2]
New Zealand	April–September	[2, 3]
Fiji	March–April, July–September, usually a single wave per year	[2]
Brazil	varies by latitude	[17]
Fortaleza, Brazil	Jan–July	[18]
Bogotá, Colombia	September–November, year-round	[19]
Chile	May–August	[2]
Panama	May–August	[2]
Nicaragua	June–July, October–December	[20]
Dakar, Senegal	June–September	[21]
Kenya	year-round, more in colder months	[22]
Cameroon	year-round, Oct-Nov peak	[23]

References

[1] Schanzer DL, Langley JM, Dummer T, Viboud C, Tam TWS (2010) A composite epidemic curve for seasonal influenza in Canada with an international comparison. Influenza Other Respi Viruses 4: 295-306.

[2] Reicheldelfer PS, Kendal AP, Shortridge KF, Hampson A (1989) Influenza surveillance in the Pacific basin: seasonability of virus occurrence. In: Chan YC, Doraisingham S, Ling AE, editors, Proceedings of First Asia-Pacific Congress of Medical Virology. World Scientific, p. 41244.

[3] Hampson AW (1999) Epidemiological data on influenza in Asian countries. Vaccine 17 Suppl 1: S19-23.

[4] Mokhtari-Azad T, Mohammadi H, Moosavi IA, Saadatmand Z, Nategh R (2004) Influenza surveillance in the Islamic Republic of Iran from 1991 to 2001. East Mediterr Health J 10: 315-21.

[5] al Hajjar S, Akhter J, al Jumaah S, Hussain Qadri SM (1998) Respiratory viruses in children attending a major referral centre in Saudi Arabia. Ann Trop Paediatr 18: 87-92.

[6] Shu YL, Fang LQ, de Vlas SJ, Gao Y, Richardus JH, et al. (2010) Dual seasonal patterns for influenza, China. Emerg Infect Dis 16: 725-6.

[7] Li C, Choi B, Wong T (2006) Influenza-related deaths and hospitalizations in Hong Kong: A subtropical area. Public Health 120: 517 - 524.

[8] Fitzner KA, McGhee SM, Hedley AJ, Shortridge KF (1999) Influenza surveillance in Hong Kong: results of a trial physician sentinel programme. Hong Kong Med J 5: 87-94.

[9] Hsieh YC, Chen HY, Yen JJ, Liu DP, Chang LY, et al. (2005) Influenza in Taiwan: seasonality and vaccine strain match. J Microbiol Immunol Infect 38: 238-43.

[10] Rao BL, Banerjee K (1993) Influenza surveillance in Pune, India, 1978–90. Bulletin of the World Health Organization 71: 177–181.

[11] Agrawal AS, Sarkar M, Chakrabarti S, Rajendran K, Kaur H, et al. (2009) Comparative evaluation of real-time PCR and conventional RT-PCR during a 2 year surveillance for influenza and respiratory syncytial virus among children with acute respiratory infections in Kolkata, India, reveals a distinct seasonality of infection. J Med Microbiol 58(Pt 12): 1616-22.

[12] Zaman RU, Alamgir ASM, Rahman M, Azziz-Baumgartner E, Gurley ES, et al. (2009) Influenza in outpatient ILI case-patients in national hospital-based surveillance, Bangladesh, 2007–2008. PLoS One 4: e8452.

[13] Nguyen HLK, Saito R, Ngiem HK, Nishikawa M, Shobugawa Y, et al. (2007) Epidemiology of influenza in Hanoi, Vietnam, from 2001 to 2003. J Infect 55: 58-63.

[14] Mardy S, Ly S, Heng S, Vong S, Huch C, et al. (2009) Influenza activity in Cambodia during 2006–2008. BMC Infect Dis 9: 168.

[15] Blair PJ, Wierzba TF, Touch S, Vonthanak S, Xu X, et al. (2010) Influenza epidemiology and characterization of influenza viruses in patients seeking treatment for acute fever in Cambodia. Epidemiol Infect 138: 199-209.

[16] Beckett CG, Kosasih H, Ma'roef C, Listiyaningsih E, Elyazar IRF, et al. (2004) Influenza surveillance in Indonesia: 1999–2003. Clinical Infectious Diseases 39: 443-449.

[17] Alonso WJ, Viboud C, Simonsen L, Hirano EW, Daufenbach LZ, et al. (2007) Seasonality of influenza in Brazil: a traveling wave from the Amazon to the subtropics. Am J Epidemiol 165: 1434-42.

[18] Moura FE, Perdigao ACB, Siqueira MM (2009) Seasonality of influenza in the tropics: a distinct pattern in northeastern Brazil. Am J Trop Med Hyg 81: 180-3.

[19] Ramírez AP, Mendoza AR, Montoya JM, Cótes K, López JD, et al. (2009) Mortalidad asociada con las temporadas de mayor circulación de los virus de la influenza en Bogotá, Colombia, 1997-2005 [Mortality associated with peak seasons of influenza virus circulation in Bogotá, Colombia, 1997–2005]. Rev Panam Salud Publica 26: 435-9.

[20] Gordon A, Ortega O, Kuan G, Reingold A, Saborio S, et al. (2009) Prevalence and seasonality of influenza-like illness in children, Nicaragua, 2005–2007. Emerg Infect Dis 15: 408-14.

[21] Dosseh A, Ndiaye K, Spiegel A, Sagna M, Mathiot C (2000) Epidemiological and virological influenza survey in Dakar, Senegal: 1996–1998. Am J Trop Med Hyg 62: 639-43.

[22] Gachara G, Ngeranwa J, Magana JM, Simwa JM, Wango PW, et al. (2006) Influenza virus strains in Nairobi, Kenya. J Clin Virol 35: 117-8.

[23] Tchidjou HK, Vescio F, Boros S, Guemkam G, Minka E, et al. (2010) Seasonal pattern of hospitalization from acute respiratory infections in Yaoundé, Cameroon. J Trop Pediatr 56: 317-20.
