# Supplementary material for: The Global Transmission and Control of Influenza
Source: PLoS One. 2011 May 6;6(5):e19515. doi: 10.1371/journal.pone.0019515 (PMC3089626; doi:10.1371/journal.pone.0019515)
Supplement: Table S3 — Cities in the 13 transmission clusters from the global model, in decreasing order of flow. (PDF) [file pone.0019515.s014.pdf]

Table S3. Cities in the 13 transmission clusters from the global model, in decreasing order of flow.

Cluster	Cities
Europe and North/West Africa	Abidjan, Accra, Aleppo, Algiers, Amsterdam, Ankara, Antananarivo, Astrakhan, Athens, Barcelona, Basel, Belfast, Belgrade, Bergen, Berlin, Birmingham, Bologna, Brussels, Bucharest, Budapest, Casablanca, Catania, Cologne, Copenhagen, Dakar, Douala, Dublin, Dusseldorf, Edinburgh, Frankfurt, Geneva, Glasgow, Gothenburg, Hamburg, Hanover, Havana, Helsinki, Istanbul, Izmir, Kinshasa, Kosice, Lagos, Las Palmas, Lisbon, Ljubljana, London, Luanda, Luxembourg, Lyon, Madrid, Malaga, Malta, Manchester, Marseille, Mauritius, Milan, Munich, Nice, Nicosia, Oslo, Palermo, Palma de Mallorca, Paris, Petrozavodsk, Prague, Reykjavik, Riga, Rome, Sofia, Stavanger, Stockholm, Stuttgart, Tallinn, Tel Aviv, Tenerife, Tirana, Toulouse, Tromso, Tunis, Turin, Venice, Vienna, Vilnius, Warsaw, Zagreb, Zurich
North/Central America and Caribbean	Atlanta, Boston, Calgary, Cancun, Caracas, Charlotte, Chicago, Cincinnati, Ciudad de Panama, Cleveland, Dallas, Denver, Detroit, Edmonton, Georgetown, Guadalajara, Guayaquil, Honolulu, Houston, Indianapolis, Kansas City, Las Vegas, Lima, Los Angeles, Majuro, Managua, Maracaibo, Memphis, Mexico City, Miami, Minneapolis, Monterrey, Montreal, Nashville, Nassau, New Orleans, New York, Orlando, Ottawa, Papeete, Philadelphia, Phoenix, Pittsburgh, Portland, Quebec, Raleigh, Salt Lake City, San Diego, San Francisco, San Jose, San Juan, San Salvador, Santo Domingo, Seattle, Sioux Lookout, St. Johns, St. Louis, Tampa, Thunder Bay, Toronto, Vancouver, Washington, Winnipeg, Yellowknife
Middle East and South Asia	Abu Dhabi, Ad-Dawhah, Addis Ababa, Ahmedabad, Amman, Bahrain, Bangalore, Cairo, Calcutta, Chennai, Chittagong, Colombo, Delhi, Dhaka, Dubai, Faisalabad, Hyderabad, Indore, Jaipur, Jeddah, Kano, Karachi, Kathmandu, Khartoum, Kuwait, Lahore, Lucknow, Mashad, Mumbai, Muscat, Nagpur, Pune, Riyadh, Tehran
Hong Kong and Southeast Asia	Bandung, Bangkok, Denpasar, Haiphong, Ho Chi Minh City, Hong Kong, Jakarta, Kaohsiung, Kuala Lumpur, Manila, Medan, Phnom Penh, Rangoon, Singapore, Surabaya, Taipei

Continued next page…

. . . continued.

**Cluster**	**Cities**
China and North Korea	Beijing, Changchun, Chengdu, Chongqing, Dalian, Guangzhou, Haikou, Hangzhou, Harbin, Jinan, Kunming, Nanjing, Pyongyang, Qingdao, Shanghai, Shenyang, Shenzhen, Shijiazhuang, Taiyuan, Tianjin, Wuhan, Xi An, Xiamen, Zhengzhou
Russia and Central Asia	Almaty, Arkhangelsk, Baku, Barnaul, Belgorod, Biskek, Chelyabinsk, Chita, Ekaterinburg, Irkutsk, Kaliningrad, Kazan, Kemerovo, Khabarovsk, Kiev, Krasnodar, Krasnoyarsk, Magadan, Minsk, Moscow, Murmansk, Nizhniy Novgorod, Norilsk, Novosibirsk, Omsk, Perm, Petropavlovsk-Kamchats, Rostov, Samara, Saratov, St. Petersburg, Stavropol, Syktyvkar, Tashkent, Tbilisi, Ufa, Ulan-Ude, Volgograd, Yakutsk, Yerevan, Yuzhno-Sakhalinsk
Japan	Fukuoka, Kagoshima, Nagoya, Okinawa, Osaka, Sapporo, Tokyo
Australia, New Zealand, and Pacific Islands	Adelaide, Auckland, Brisbane, Cairns, Christchurch, Darwin, Honiara, Lae, Melbourne, Nadi, Perth, Port Moresby, Port Vila, Sydney, Wellington
South America	Asuncion, Belo Horizonte, Brasilia, Buenos Aires, Fortaleza, Manaus, Montevideo, Rio de Janeiro, Salvador, Santa Cruz de la Sierra, Santiago, Sao Paulo
Southern/Eastern Africa	Blantyre, Cape Town, Dar es Salaam, Durban, Gaborone, Harare, Johannesburg, Kampala, Lusaka, Maputo, Maseru, Nairobi
South Korea and Mongolia	Cheju, Pusan, Seoul, Taegu, Ulaanbaatar
Colombia	Bogota, Cali, Medellin
Greenland	Kangerlussuaq, Nuuk
