# Supplementary material for: The Global Transmission and Control of Influenza
Source: PLoS One. 2011 May 6;6(5):e19515. doi: 10.1371/journal.pone.0019515 (PMC3089626; doi:10.1371/journal.pone.0019515)
Supplement: Table S4 — Observed and simulated pandemic H1N1 2009 epidemic peaks. Observed data was from influenza A virology surveillance data from Flunet. (PDF) [file pone.0019515.s015.pdf]

Table S4. Observed and simulated pandemic H1N1 2009 epidemic peaks.

Country	Observed (week)[1]	Observed (date of mid-week)[1]	Best-fit model peak
Argentina	27	7/8	6/29
Australia	30	7/29	7/12
Canada	45	11/11	11/23
Chile	28	7/15	6/27
France	48	12/2	11/14
Greece	48	12/2	11/17
Italy	46	11/18	11/11
Japan	44	11/4	11/6
New Zealand	28	7/15	7/6
Norway	45	11/11	11/10
Philippines	26	7/1	7/21
Romania	48	12/2	11/28
Russia	48	12/2	12/4
South Korea	44	11/4	11/5
Turkey	47	11/25	11/20
United States of America	43	10/28	11/23

[1]Observed data was from influenza A virology surveillance data from Flunet.
