# Supplementary material for: The Global Transmission and Control of Influenza
Source: PLoS One. 2011 May 6;6(5):e19515. doi: 10.1371/journal.pone.0019515 (PMC3089626; doi:10.1371/journal.pone.0019515)
Supplement: Table S5 — Observed and simulated 1968–1969 pandemic peaks. Observed data is from [3]. (PDF) [file pone.0019515.s016.pdf]

Table S5. Observed and simulated 1968–1969 pandemic peaks.

Country	Epidemic peak [1]	Best-fit model peak
Hong Kong	7/25/1968	7/22/1968
Los Angeles	12/20/1968	1/25/1969
New York	12/30/1968	1/2/1969
London	1/21/1969	1/19/1969
Paris	1/21/1969	1/21/1969
Tokyo	1/21/1969	1/3/1969
Sydney	8/5/1969	8/30/1969

[1]Rvachev LA, Longini IM Jr (1985) A mathematical model for the global spread of influenza. Mathematical Biosciences 75: 3–22.
