# Supplementary material for: The Global Transmission and Control of Influenza
Source: PLoS One. 2011 May 6;6(5):e19515. doi: 10.1371/journal.pone.0019515 (PMC3089626; doi:10.1371/journal.pone.0019515)
Supplement: Table S6 — Vaccine availability in different countries in the model. (PDF) [file pone.0019515.s017.pdf]

Table S6. Vaccine availability in different countries in the model.

Coverage	Countries
High	Australia, Austria, Bahrain, Belgium, Canada, Denmark, Finland, France, Germany, Greenland, Hong Kong, Iceland, Ireland, Japan, Kuwait, Luxembourg, Monaco, Netherlands, New Zealand, Norway, Qatar, Saudi Arabia, Singapore, Sweden, Switzerland, United Arab Emirates, United Kingdom, United States
Medium	Argentina, Bahamas, Belarus, Botswana, Chile, Colombia, Croatia, Cyprus, Czech Republic, Estonia, Greece, Hungary, Israel, Italy, Kazakhstan, Latvia, Libya, Lithuania, Malaysia, Malta, Mauritius, Oman, Poland, Portugal, Russia, Serbia, Slovakia, Slovenia, South Korea, Spain, Taiwan, Uruguay
Low	Afghanistan, Albania, Algeria, Angola, Armenia, Azerbaijan, Bangladesh, Bolivia, Brazil, Bulgaria, Burma, Cambodia, Cameroon, China, Costa Rica, Côte d'Ivoire, Cuba, Dominican Republic, Democratic Republic of Congo, Ecuador, Egypt, El Salvador, Ethiopia, Fiji, French Polynesia, Georgia, Ghana, Guatemala, Guyana, India, Indonesia, Iran, Jordan, Kenya, Kyrgyzstan, Lebanon, Lesotho, Liberia, Madagascar, Malawi, Mali, Marshall Islands, Mauritania, Mexico, Mongolia, Morocco, Mozambique, Nepal, Nicaragua, Nigeria, North Korea, Pakistan, Panama, Papua New Guinea, Paraguay, Peru, Philippines, Romania, Senegal, Solomon Islands, Solomon Islands, South Africa, Sri Lanka, Sudan, Syria, Tanzania, Thailand, Tunisia, Turkey, Uganda, Ukraine, Uzbekistan, Vanuatu, Venezuela, Vietnam, Yemen, Zambia, Zimbabwe
