# Supplementary material for: The Global Transmission and Control of Influenza
Source: PLoS One. 2011 May 6;6(5):e19515. doi: 10.1371/journal.pone.0019515 (PMC3089626; doi:10.1371/journal.pone.0019515)
Supplement: Text S1 — Model. (PDF) [file pone.0019515.s018.pdf]

The global transmission and control of influenza: Supporting Text 1

Eben Kenah[1,2,†], Dennis L. Chao[1,†,*], Laura Matrajt[4], M. Elizabeth Halloran[1,3], and Ira M. Longini, Jr.[1,3]

[1]Center for Statistics and Quantitative Infectious Diseases
Vaccine and Infectious Disease Division
Fred Hutchinson Cancer Research Center, Seattle, Washington, USA

[2]Department of Biostatistics, School of Public Health
University of Washington, Seattle, Washington, USA

[3]Department of Applied Mathematics
University of Washington, Seattle, Washington, USA

† These authors contributed equally to the work.
∗ E-mail: dchao@fhcrc.org

1 Model

Here, we describe a new global model of influenza pandemics. The model includes 321 major cities on six continents, the airline travel among them, and influenza seasonality data when available. This model includes more detailed within-host influenza dynamics, more detailed influenza vaccine behavior, and more detailed seasonality data for tropical regions than other models. Nonetheless, it is a relatively simple model that does not require specialized computing resources to use. The model has two layers: a set of within-city models and a global model that links them through the air transportation network and controls seasonality. Parameters are summarized in Table S1. We first describe the within-city model.

1.1 Within-city model

1.1.1 Population, disease, and compartments

Subpopulations and risk groups Within each city, susceptibles are divided into *subpopulations* and *risk groups*. Subpopulation membership is used to determine incoming and outgoing infection probabilities. Membership in risk groups is used to determine morbidity and mortality. At initialization, the argument `propList` is a list that gives the proportion of the population in

each subpopulation, and the argument `riskProps` is a nested list in which the jth element of the ith list gives the proportion of subpopulation i in risk group j. These arguments are used to generate two arrays, `propArray` and `popArray` whose $(i, j)^{\text{th}}$ elements are the proportion of the population and the number of persons, respectively, in subpopulation i and risk group j.

Natural history of infection Upon infection, each person is first assigned to become asymptomatic or symptomatic. He or she is then assigned uniformly at random to one of the six viral load trajectories shown in Figure S1. On all trajectories, infection lasts six days. At initialization, the argument `psymp` is the probability that an unvaccinated person becomes symptomatic. Vaccination can reduce the probability of becoming symptomatic. The ratio of asymptomatic infectiousness to symptomatic infectiousness is given by the argument `m`. In our simulations, $\texttt{psymp} = \frac{2}{3}$ and $m = 0.5$.

Susceptibles The population in each city is divided into susceptible (`S`), infected (`I`), and removed (`R`) compartments. There are two representations of susceptible persons in the model: `S` and `Sdict`. `S` is an $s \times r \times 2$ NumPy array, where s is the number of subpopulations and r is the number of risk groups. $\texttt{S}[i, j, k]$ is the number of persons in subpopulation i, risk group j, and binary vaccination status k (0 for unvaccinated, 1 for vaccinated). The dictionary `Sdict` maps each vaccination time `vtimeS` to an $s \times r$ NumPy array representing the number of persons in each subpopulation/risk group combination vaccinated at time `vtimeS`. The array containing unvaccinated persons is associated with the key `None`. `Sdict` is used to implement time-dependent effects of vaccination.

Infecteds There are three representations of infected persons in the model: `incI`, `I`, and `Idict`. `incI` is an $s \times r \times 2$ NumPy array where $\texttt{incI}[i, j, k]$ is the number of person in subpopulation i, risk group j, and binary vaccination status k who are in the first day of infection. `I` is a NumPy vector of length s whose i^{th} element is the number of infected persons in subpopulation i. The dictionary `Idict` maps each vaccination time `vtimeI` to an $s \times 2 \times 6 \times 6$ NumPy array whose $(i, j, v, d)^{\text{th}}$ element is the number of infected persons in subpopulation i with binary symptom status j and viral load trajectory v on day $d + 1$ of infection who were vaccinated at time `vtimeI`. The array containing unvaccinated persons has the key `None` and that containing persons vaccinated ≥ 28 has the key `'Past'`.

Recovereds There are two representations of recovered persons in the model: `R` and `Rq`. `R` is an $s \times r \times 2$ NumPy array where $\texttt{R}[i, j, k]$ is the number of recovered persons in subpopulation i, risk group j, and binary vaccination status k. `Rq` is a first-in-first-out (FIFO) queue of length 6 where $\texttt{Rq}[d]$ is an $s \times r \times 2$ NumPy array representing persons in the $d + 1^{\text{st}}$ day of illness. This allows persons to be added to the appropriate subpopulation and risk group in `R` without keeping track of risk groups in the infected compartments, making the calculation of infection

probabilities more efficient. It is assumed that vaccination has an effect only prior to infection, so the binary vaccination status is fixed upon infection.

Data storage The within-city model stores data in five lists: `tlist`, `Slist`, `incIlist`, `Ilist`, and `Rlist`. Each list keeps a copy of the corresponding compartment for each time included in `tlist`. To save memory, copies of `Sdict`, `Idict`, and `Rq` are not retained.

1.1.2 Transmission of infection

Transmission of disease is governed by a matrix `betaMatrix` and a parameter `m` representing the ratio of asymptomatic to symptomatic infectiousness. We first describe the calculation of infection probabilities in the absence of vaccination and seasonality. We then discuss the implementation of seasonality and the effects of vaccination.

Transmission parameters `betaMatrix` is an $s \times s$ matrix whose $(i, j)^{\text{th}}$ element is the probability per unit viral load that an unvaccinated symptomatic infectious person in subpopulation i infects an unvaccinated susceptible person in subpopulation j in one time step. `betaMatrix` can be specified as an argument at initialization of the within-city model. More commonly, it is calculated from the arguments `R0` and `NGmatrix`. `R0` is the desired within-city R_0. `NGmatrix` is the within-city next-generation matrix, whose $(i, j)^{\text{th}}$ element is the expected number of secondary infections in subpopulation j produced by an infected person from subpopulation i, assuming subpopulation j is completely susceptible. If exactly one of `betaMatrix` or `NGmatrix` is specified, the other will be calculated. If `R0` is not specified, it will be set equal to the spectral radius of `NGmatrix`. If `R0` is specified, `NGmatrix` and `betaMatrix` will be scaled to obtain a spectral radius for `NGmatrix` equal to `R0`.

Seasonality Seasonality is implemented by varying the within-city R according to the day of the year and the location of the city. Before calculating transmission probabilities each day, the `betaMatrix` of each city is multiplied by its `R0ratio`.

Infection escape probabilities Recall that `m` gives the relative infectiousness of an asymptomatic case compared to a symptomatic case. Let v_d be the viral load of trajectory v on day d (in $\log_{10}$ TCID_{50}/ml), and let $\mathsf{VE_S}$ and $\mathsf{VE_I}$ be the vaccine efficacies for susceptibility and infectiousness, respectively. Let $p_{ijvd,s}(\tau_{\text{inf}}, \tau_{\text{sus}})$ be the probability per day that a susceptible in subpopulation s vaccinated τ_{sus} days ago will be infected by an infected person in subpopulation i with symptom status j and viral load trajectory v on day d of illness who was vaccinated τ_{inf}

days ago. Then

$$
\begin{aligned}
p_{ijvd,s}(\texttt{None},\texttt{None}) &= \texttt{m}^{1-j} v_d \beta_{is} & (1)\\
p_{ijvd,s}(\texttt{None},\tau_{\text{sus}}) &= \big(1-\mathsf{VE}_{\mathsf{S}}(\tau_{\text{sus}})\big)\texttt{m}^{1-j} v_d \beta_{is} & (2)\\
p_{ijvd,s}(\tau_{\text{inf}},\texttt{None}) &= \big(1-\mathsf{VE}_{\mathsf{I}}(\tau_{\text{inf}})\big)\texttt{m}^{1-j} v_d \beta_{is} & (3)\\
p_{ijvd,s}(\tau_{\text{inf}},\tau_{\text{sus}}) &= \big(1-\mathsf{VE}_{\mathsf{I}}(\tau_{\text{inf}})\big)\big(1-\mathsf{VE}_{\mathsf{S}}(\tau_{\text{sus}})\big)\texttt{m}^{1-j} v_d \beta_{is}, & (4)
\end{aligned}
$$

where $\tau = \texttt{None}$ indicates an unvaccinated person and $\beta_{is} = \texttt{betaMatrix}[i,s]$. Fix a day and let $I_{ijvd}(\tau)$ denote the number of infected persons in subpopulation i with symptom status j and viral load trajectory v on day d of illness who were vaccinated τ days ago. Then a susceptible person in subpopulation s who was vaccinated τ_{sus} days ago escapes infection on that day with probability

$$
Q_s(\tau_{\text{sus}}) = \prod_{\tau,ijvd} \big(1 - p_{ijvd,s}(\tau,\tau_{\text{sus}})\big)^{I_{ijvd}(\tau)}. \qquad (5)
$$

After infection, an unvaccinated susceptible becomes symptomatic with probability `psymp` and a vaccinated susceptible becomes symptomatic with probability $\mathsf{VE}_{\mathsf{P}}(\tau_{\text{sus}}) \times \texttt{psymp}$.

Transition from S to I At time t, let $S_i(\tau)$ be the number of susceptibles in subpopulation i who were vaccinated τ days ago (with $\tau = \texttt{None}$ for unvaccinated susceptibles, and let $I_i^*(\tau)$ be the number of new infections among them. Then

$$
I_i^*(\tau) \sim \text{binomial}\big(S_i(\tau_{\text{sus}}), 1 - Q_i(\tau)\big), \qquad (6)
$$

where $S_i(\tau) = \texttt{Sdict}[t-\tau][i]$ and $Q_i(\tau)$ is given in equation (5). To reduce the number of infected compartments in the model, we have new infections become symptomatic or asymptomatic immediately. Let $I_{i0}^*(\tau)$ and $I_{i1}^*(\tau)$ be the numbers of symptomatic and asymptomatic infections among the $I_i^*(\tau)$ infections from subpopulation i vaccinated at time $t-\tau$. Then

$$
\begin{aligned}
I_{i0}^*(\tau) &= I_i^*(\tau) - I_{i1}^*(\tau) & (7)\\
I_{i1}^*(\tau) &\sim \text{binomial}\Big(I_i^*, \big(1-\mathsf{VE}_{\mathsf{P}}(\tau)\big)\texttt{psymp}\Big) & (8)
\end{aligned}
$$

Each newly infected person is assigned to one of six viral load trajectories, with probability $\frac{1}{6}$ of being assigned to each trajectory.

1.1.3 Implementation

The model is written in Python 2.6.2 (`http://www.python.org`) using the SciPy and NumPy packages [1]. The within-city model is implemented in an object called an `epiList`, which contains all of the functions and data required to run the model. A within-city epidemic can be run using the function `city_epidemic`. The primary functions of the within-city model are:

imprt converts a number of infections to import into an infection probability for susceptibles. Infected susceptibles are sent to the appropriate infected compartments, with new infected compartments created as necessary.

vaccinate vaccinates each unvaccinated susceptible or infected person with a given probability. This probability can vary by city, subpopulation, and risk group, and it can depend on the time and the current state of the model. Vaccinated persons are placed in new vaccinated susceptible and infected compartments associated with the vaccination time.

increment calculates infection probabilities, chooses newly infected persons from each susceptible compartment, subdivides them by symptom status and viral load trajectory, and places them in the appropriate infected compartment, creating new compartments as necessary. It also places susceptible and infected compartments associated with vaccination times ≥ 28 days ago into the corresponding `'Past'` compartments.

Each of these is allowed at most once in each time step; only `increment` is required. Within each time step, they must occur in the following order: (1) `imprt`, (2) `vaccinate`, and (3) `increment`. At the end of each time step, the number of susceptible, infectious, and recovered persons is recorded.

1.2 Multi-city (global) model

The global model links together the 321 within-city models by allowing travel between them and controls seasonality. Locations of the cities are shown in Figure S2. The model is based on [2] and uses air transport data from [3]. It records the total number of susceptibles, incident infections, infections, and recovereds in each time step, and (optionally) can store a matrix for each time step showing the number of travelers from each city to every other city.

1.2.1 Age groups

All cities are divided into two age groups: 0–14 years old and 15+ years old. The population of each city is divided into two age groups based on the proportion of the population under 15 in its country [4]. The choice of 15 years as a cutoff was made because it is relevant to the transmission of influenza and we have data about the proportion of the national population in these age categories for each nation containing at least one of the 321 cities in the model. (We also have information about the proportion of national populations above 65 years old, but these people are currently grouped with everyone else over 15.) The transmission probabilities are tuned to obtain a next-generation matrix proportional to:

		Mean secondary cases	
		Children	Adults
Primary case	Child	1.8	0.5
	Adult	0.5	0.2

(9)

from page 4 of [5]. This next-generation matrix is constant across cities regardless of age distribution. We experimented with having a scaled transmission-probability matrix and allowing the next-generation matrix to vary with the age distribution, but this produced unrealistically low R_0 in Europe and Japan and unrealistically high R_0 in Africa and the Middle East.

1.2.2 Travel between cities

For efficiency, only infected travelers are tracked in the model. Infected visitors to a city are put into the infected compartment corresponding to their vaccination time, symptom status, viral load trajectory, and day of illness. They progress through the infected compartments and travel to other cities just like the other infected persons in the destination city. Upon recovery, they return immediately to their home city.

Departures Let V_{AB} be the number of persons from city A who travel to city B per day. In each time step, each asymptomatic infected person in city A travels travels to city B with probability

$$P_{AB} = \frac{V_{AB}}{\mathrm{Pop}_A}, \tag{10}$$

where Pop_A is the population of city A (residents only). All persons who depart city A for city B at time t spend all of day t in city A and all of day $t+1$ in city B. Symptomatic infected persons travel from A to B with probability $\texttt{sympTratio} \times P_{AB}$, where `sympTratio` is an attribute of the within-city model. In the global model, all cities have the same value of `sympTratio`, which can be set to zero for consistency with [2] and other prior work. In our simulations, we use $\texttt{sympTratio} = 0.25$. The results are most sensitive to this ratio in less connected regions, where the pandemic might peak during the second season (Figure S4).

Arrivals Upon arrival, each visitor is placed in the infected compartment that corresponds to his or her vaccination time in the destination city. If no such compartment exists, a new one is created. Within that compartment, he or she is placed in the appropriate symptom status, viral load trajectory, and day of illness.

1.2.3 Implementation

The global model is implemented through a `globalModel` object. Implementing travel between cities and seasonality required a few additions to the `epiList` objects used for the within-city models.

Additions to within-city models The `send` function creates a list of tuples consisting of vaccination times and $2 \times 2 \times 6 \times 6 \times k$ NumPy matrices of infected visitors going to other cities, where k is the number of destination cities to which at least one infected person is traveling. The cities to which people can travel are kept in a list called `destList` and the probability

of traveling to each destination is kept in a list called `destProbs`, whose last entry is the probability of remaining in the city. The `receive` function takes a list of tuples containing vaccination times and $2 \times 2 \times 6 \times 6$ NumPy matrices of infected arrivals and adds them to the appropriate infected compartments. The `exchange` function of `globalModel` runs the `send` function of each city with at least one infected person and distributes the visitors to destination cities through their `receive` functions. The list `Rq` of each city ensures that infected residents are returned to the appropriate recovered compartment of their home city upon recovery from infection.

Functions A global epidemic is run using the function `global_epidemic`. The primary functions in the `globalModel` are

`exchange` exchanges visitors between cities using their `send` and `receive` functions.

`imprt` imports infections to specified cities using their `imprt` functions.

`vaccinate` vaccinates persons in specified cities using their `vaccinate` functions..

`increment` adjusts the `R0ratio` for all cities and increments the model by calling their `increment` functions. It records the total number of susceptibles, new infections, infecteds, and recovereds in the model. Optionally, it can also record a matrix of numbers of travelers between each ordered pair of cities.

Each function can be called only once in each time step; only `exchange` and `increment` are required. Within each time step, they must be called in the following order: (1) `exchange`, (2) `imprt`, (3) `vaccinate`, and (4) `increment`. In the base class, `globalModel`, there is no seasonality and no vaccination. Seasonality and vaccination strategies are implemented as subclasses.

Data The `epiList` for each city is kept in a dictionary called `Cities` that maps city names to the corresponding `epiList` objects. The total number of susceptibles, new infections, infecteds, and recovereds in the global model are kept in variables called `S`, `I`, and `R`. The time steps from the model are kept in the list `tlist`, and lists of the number of susceptibles, incident infections, infecteds, and recovereds at each time step in each city are kept in lists `Slist`, `incIlist`, `Ilist`, and `Rlist`. (The corresponding lists for each city are attributes of the corresponding `epiList` object, as described above.) If `exchangeList == True`, the global model will save a matrix of the number of travelers in each (origin, destination) pair at each time step.

References

[1] Jones E, Oliphant T, Peterson P, et al. (2001–2009). SciPy: Open source scientific tools for Python. URL http://www.scipy.org/.

[2] Rvachev LA, Longini IM Jr (1985) A mathematical model for the global spread of influenza. Mathematical Biosciences 75: 3–22.

[3] Epstein JM, Goedecke DM, Yu F, Morris RJ, Wagener DK, et al. (2007) Controlling pandemic flu: the value of international air travel restrictions. PLoS One 2: e401.

[4] Central Intelligence Agency (2009) The World Factbook 2009. Washington, DC: Central Intelligence Agency.

[5] McBryde ES, Bergeri I, van Gemert C, Rotty J, Headley EJ, et al. (2009) Early transmission characteristics of influenza A(H1N1)v in Australia: Victorian state, 16 May – 3 June 2009. Euro Surveill 14: 19363.
