# Supplementary material for: The Global Transmission and Control of Influenza
Source: PLoS One. 2011 May 6;6(5):e19515. doi: 10.1371/journal.pone.0019515 (PMC3089626; doi:10.1371/journal.pone.0019515)
Supplement: Text S2 — Mapping global influenza transmission. (PDF) [file pone.0019515.s019.pdf]

The global transmission and control of influenza: Supporting Text 2

Eben Kenah[1,2,†], Dennis L. Chao[1,†,*], Laura Matrajt[4], M. Elizabeth Halloran[1,3], and Ira M. Longini, Jr.[1,3]

[1]Center for Statistics and Quantitative Infectious Diseases
Vaccine and Infectious Disease Division
Fred Hutchinson Cancer Research Center, Seattle, Washington, USA

[2]Department of Biostatistics, School of Public Health
University of Washington, Seattle, Washington, USA

[3]Department of Applied Mathematics
University of Washington, Seattle, Washington, USA

† These authors contributed equally to the work.
∗ E-mail: dchao@fhcrc.org

2 Mapping global influenza transmission

The epidemic percolation network (EPN) is a directed random network that represents the final outcomes of a stochastic epidemic model [1]. To generate an EPN, we imagine that every individual in the model is infected and draw arrows from each individual i to all persons with whom i would make infectious contact while he or she was infected. We define infectious contact to be a contact sufficient to infect a susceptible person, so an arrow from i to j means that infection of i implies infection of j, either by i or by someone who makes infectious contact with j before i does. An epidemic begins with one or more persons infected from outside the population, who we call *imported infections*. For a given set of imported infections and a given realization of the EPN, the set of people who will eventually be infected is precisely the union of the out-components of all imported infections. Since infectious contact is a stochastic process, the EPN is a random network. It can be shown that the size distribution of outbreaks starting with the infection of a node i is identical to the distribution of out-component sizes of i in the probability space of EPNs. In the limit of a large population, the epidemic threshold corresponds to the emergence of a giant strongly-connected component in the EPN, the probability of a large epidemic given a single randomly-chosen initial infection is equal to the proportion of the nodes contained in the giant in-component of the EPN, and the attack rate of a large epidemic

(for any set of initial infections) is equal to the proportion of nodes contained in the giant out-component [1, 2].

To understand the global spread of influenza in our model, we made a simplified map of the EPN it generates. Instead of dealing directly with a realization of the EPN, we constructed a city-to-city transmission network, where each node represents a city and there is a directed edge from each city i to all cities to which people can travel directly from i. Each edge ij was then assigned a weight proportional to the expected number of edges from persons in city i to persons in city j in the EPN, assuming all cities are transmitting at the peak seasonal R_0. This results in a network with 321 nodes and 53,354 weighted edges. This network was produced in Python 2.6 using the NetworkX package [3], available at `http://networkx.lanl.gov`.

Let `sympr` be the mean number of secondary infections generated each day by a symptomatic infectious person in city i, and let p_C and p_A denote the proportion of children and adults city i. Then

$$\texttt{sympr} = \frac{1}{6}\begin{bmatrix} p_C \\ p_A \end{bmatrix} \cdot \texttt{NGmatrix}\begin{bmatrix} 1 \\ 1 \end{bmatrix}, \tag{1}$$

and $\texttt{asympr} = \texttt{m} \times \texttt{sympr}$, where m_i is the ratio of asymptomatic to symptomatic infectiousness. Now let $\texttt{sympP}_{ij}$ be the per-day probability that a symptomatic person in city i travels to city j, so $\texttt{asympP}_{ij} = \texttt{sympTratio} \times \texttt{sympP}_{ij}$ is the per-day probability that an asymptomatic person in city i travels to city j. Then the weight of the edge from city i to city j is

$$\sum_{k=0}^{5} (6-k)\big[(1-\texttt{psymp}) \times (1-\texttt{asympP}_{ij})^k \texttt{asympP}_{ij} \times \texttt{asympr} + \texttt{psymp} \times (1-\texttt{sympP}_{ij})^k \texttt{sympP}_{ij} \times \texttt{sympr}\big]. \tag{2}$$

Since `sympr` and `asympr` vary slightly by city, this approximates the true expected number of edges from i to j in the probability space of city-to-city EPNs. Recall that in our simulations we use $\texttt{psymp} = \frac{2}{3}$ and $\texttt{m} = 0.5$.

2.1 Identifying transmission clusters

To simplify this map, we used an information-theoretic clustering algorithm based on using a two-level code to describe the path of a random walker [4]. In a one-level code, each node in the network must be given a unique name, so an efficient code will assign short code names to frequently visited nodes and longer names to rarely visited nodes. In a two-level description, nodes are grouped into clusters. Each cluster is assigned a unique code, and nodes within clusters are assigned short codes that can be reused in different clusters, like city names within states. If clusters can be assigned such that a random walker is likely to take many steps within a cluster before leaving it, this two-level code will be shorter than the most efficient one-level code. The clustering algorithm searches through possible partitions of the nodes into clusters to find one that (approximately) minimizes the expected code length required to describe the path of a random walker.

In the random walk on the city-to-city EPN, the edge weights from city i to its neighbors are normalized to get transition probabilities. Since the edge weights are proportional to R_0, the random walk and the resulting clusters are independent of R_0. Since seasonality is implemented by changing the R_0 within each city, the clustering algorithm cannot capture the effects of seasonality. To ensure that the random walk approaches a unique steady state, there is a *teleportation probability* $\tau = 0.15$ that the random walker jumps to a randomly chosen city on each step. Cluster assignment is insensitive to τ unless it is very high [4].

The clustering algorithm was run and the map was produced using the Map Generator software package at `http://www.mapequation.org` [5]. The area of each cluster is proportional to the steady-state proportion of steps during which a random walker on the city-to-city EPN is in it. The proportion of the cluster area contained in the border ring is equal to the steady-state probability that a random walker inside the cluster jumps to a city in a different cluster (excluding teleportation), and the proportion of the cluster area in the interior is equal to the steady-state probability that a random walked in the cluster jumps to another city in the cluster (excluding teleportation). The width of each edge is proportional to the steady-state proportion of jumps between clusters that cross it (excluding teleportation). To make differences in area and width easier to see, the color of the interiors and border rings of the clusters get darker as their areas increase, and the colors of the edges get darker as their width increases. The cities included in each cluster are shown in Table S3, where they are listed in decreasing order of flow.

Potentially, the clustering algorithm could be made sensitive to the R_0 and population in each city by allowing the teleportation probability to depend on R_0 and having teleporters choose cities with a probability proportional to their population. Improving the identification of transmission clusters and better understanding their use in designing vaccination strategies are important extensions of the research presented here.

References

[1] Kenah E, Robins JM (2007) Second look at the spread of epidemics on networks. Physical Review E 76: 036113.

[2] Kenah E, Robins JM (2007) Network-based analysis of stochastic SIR epidemic models with random and proportionate mixing. J Theor Biol 249: 706-22.

[3] Hagberg AA, Schult DA, Swart PJ (2008) Exploring network structure, dynamics, and function using NetworkX. In: Proceedings of the 7th Python in Science Conference (SciPy2008). Pasadena, CA USA, pp. 11–15.

[4] Rosvall M, Bergstrom C (2008) Maps of random walks on complex network reveal community structure. Proc Natl Acad Sci U S A 105: 1118-1123.

[5] Edler D, Rosvall M (2010). The map generator software package. doi: `http://www.mapequation.org`.
