# Supplementary material for: The Global Transmission and Control of Influenza
Source: PLoS One. 2011 May 6;6(5):e19515. doi: 10.1371/journal.pone.0019515 (PMC3089626; doi:10.1371/journal.pone.0019515)
Supplement: Text S3 — Pandemic simulations. (PDF) [file pone.0019515.s020.pdf]

The global transmission and control of influenza: Supporting Text 3

Eben Kenah[1,2,†], Dennis L. Chao[1,†,*], Laura Matrajt[4], M. Elizabeth Halloran[1,3], and Ira M. Longini, Jr.[1,3]

[1]Center for Statistics and Quantitative Infectious Diseases
Vaccine and Infectious Disease Division
Fred Hutchinson Cancer Research Center, Seattle, Washington, USA

[2]Department of Biostatistics, School of Public Health
University of Washington, Seattle, Washington, USA

[3]Department of Applied Mathematics
University of Washington, Seattle, Washington, USA

† These authors contributed equally to the work.
∗ E-mail: dchao@fhcrc.org

3 Pandemic simulations

We first demonstrate that our model can qualitatively match the dynamics of the 1968–1969 and the 2009–2010 influenza pandemics. Then, we model the effect of a global influenza vaccination campaign on a pandemic. For all runs, we assume that symptomatic individuals have their probability of travel reduced by 75%. This is to account for individuals who are going to be symptomatic and those who travel despite being symptomatic. Australia is not very well-connected to the rest of the world, so it is sensitive to parameters like this.

The timing of epidemics for large countries is difficult to define. The model includes only major cities, so we define the peak of the country to be the average of the peaks of a country's cities. These major cities will be the first to be hit by a new epidemic strain, and in reality it will take time for the epidemic to spread to a country's smaller cities. We do not report country-wide peaks for countries that contain a wide variety of climates and influenza seasons, such as India and China

We fit the model to the epidemic peak times of pandemic H1N1 in 2009. We defined the peak of a country's pandemic H1N1 activity to be the week which had the largest number of samples positive for influenza A between March and December of 2009. The data were downloaded from http://gamapserver.who.int/GlobalAtlas/home.asp on September 13, 2010.

We used data only from countries with more than 5,000 samples in 2009 and 2010, and excluded data from China, which we believe to be too large and heterogeneous to have a single meaningful epidemic peak. The 16 countries and their observed epidemic peak times are listed in Table S4.

We assumed that the pandemic began in Mexico City (starting with 1,000 infected individuals), and simulated epidemics with different start dates and R_0. We used the difference between the observed and simulated peak times for the 16 countries with sufficient data as our error metric. We used the χ-squared statistic, $\sum \frac{(E_I - O_I)^2}{E_I}$ and found that the χ-square test using 14 degrees of freedom accepted a wide range of values for both variables (Figure S5, top panel). Because the epidemic peak times are not independent (large clusters of countries behave like a single unit, see Figure 2), the acceptance region is overly inclusive. However, the region with the highest p-values, R_0=1.6–1.95 and an epidemic start date of mid to late March, agrees with other estimates for pandemic H1N1 [1, 2, 3]. The best fit was for R_0=1.85 and the epidemic starting on March 29. (Figure S5, bottom panel).

The model results for pandemic H1N1 were in good qualitative agreement with observations. Countries in the temperate northern hemisphere peaked early in the first season (November 2009), while those in the southern hemisphere peaked during the (southern) winter of the first year (June–July 2009). There were noticeable discrepancies between the model results and observations. In the model, Mexico was predicted to have its largest epidemic peak in the spring of 2009, while it in fact the largest peak occurred in the winter of 2009–2010. This may have been due to their extensive pandemic control measures in the spring. Peak times in the United States were predicted to occur later than they actually did. The influenza season starts on September 15 in our model, based on the results in [4], but influenza activity in fact likely began in late August, when schools began to open [5].

References

[1] Fraser C, Donnelly CA, Cauchemez S, Hanage WP, Van Kerkhove MD, et al. (2009) Pandemic potential of a strain of influenza A (H1N1): Early findings. Science 324: 1557-1561.

[2] Yang Y, Sugimoto JD, Halloran ME, Basta NE, Chao DL, et al. (2009) Transmissibility and control of pandemic influenza A (H1N1) virus. Science 326: 729-33.

[3] Balcan D, Hu H, Gonçalves B, Bajardi P, Poletto C, et al. (2009) Seasonal transmission potential and activity peaks of the new influenza A(H1N1): a Monte Carlo likelihood analysis based on human mobility. BMC Med 7: 45.

[4] Schanzer DL, Langley JM, Dummer T, Viboud C, Tam TWS (2010) A composite epidemic curve for seasonal influenza in Canada with an international comparison. Influenza Other Respi Viruses 4: 295-306.

[5] Chao DL, Halloran EM, Longini IM Jr (2010) School opening dates predict pandemic influenza A(H1N1) outbreaks in the United States. J Infect Dis 202: 877-80.
